# Synthesis of Flexible Polyamide Aerogels Cross-Linked with a Tri-Isocyanate

**DOI:** 10.3390/gels10080519

**Published:** 2024-08-07

**Authors:** Daniel A. Scheiman, Haiquan Guo, Katherine J. Oosterbaan, Linda McCorkle, Baochau N. Nguyen

**Affiliations:** 1 Universities Space Research Association, NASA Glenn Research Center, 21000 Brookpark Road, Cleveland, OH 44135, USA; 2 NASA Glenn Research Center, 21000 Brookpark Road, Cleveland, OH 44135, USA

**Keywords:** flexible polyamide aerogel, tri-isocyanate cross-linker, mesoporous, density vs. modulus, thermal stability

## Abstract

A new series of flexible polyamide (PA) aerogels was synthesized using terephthaloyl chloride (TPC), 2,2′-dimethylbenzidine (DMBZ) and cross-linked with an inexpensive, commercially available tri-isocyanate (Desmodur N3300A) at polymer concentrations of 6–8 wt.% total solids and repeating units, *n*, from 30 to 60. The cross-linked DMBZ-based polyamide aerogels obtained, after supercritically drying using liquid CO_2_, had shrinkages of 19–27% with densities ranging from 0.12 g/cm^3^ to 0.22 g/cm^3^, porosity and surface areas up to 91% and 309 m^2^/g, respectively, and modulus values ranging from 20.6 to 109 MPa. Evidence suggests that a higher flexibility could be achieved using DMBZ in the polyamide backbone with N3300A as a cross-linker, when compared to previously reported TPC-mPDA-BTC PA aerogels, N3300A-polyimide aerogels, and N3300-reinforced silica aerogels.

## 1. Introduction

High-temperature aromatic polyimide and aromatic polyamide (PA) aerogels have attracted a lot of attention [1] over the last decade due to their lightweight, high porosity, above 80% in air, and high surface areas (up to 1000 m^2^/g) [2,3]. Desired properties such as infrared stealth [4,5], air filtration [6], low dielectric constant [7], separators for batteries [8,9], flame resistance [10], solar energy [11], and thermal insulation [12,13,14] can be tailored by adjusting their formulations. These aerogels can be conveniently fabricated using sol–gel technology. Their gelation time can be controlled by varying the ratio of monomers, with or without cross-linkers [15], polymer concentration, repeating units (*n*), solvents, and/or reaction conditions [16]. Despite their similarity in high mechanical strength, high-temperature use, and ease of processability, intensive studies have mostly focused on cross-linked polyimide aerogels. With a wide selection of diamine and dianhydride precursors, the rigidity or flexibility of polyimide backbones can be tuned toward certain applications. Moreover, different cross-linkers can also have tremendous effects on the overall three-dimensional structures of the polyimide aerogels. For example, polyimide aerogels composed of 3,3′,4,4′-biphenyltetracarboxylic dianhydride (BPDA) and 2,2′-dimethylbenzidine (DMBZ), at 10 wt.% with n of 20–30, cross-linked with either 1,3,5-triaminophenoxybenzene (TAB) [17], 1,3,5-benzenetricarbonyl trichloride (BTC) [18], or hexamethylene diisocyanate (or HDI trimer Desmodur N3300A) [19], exhibited relatively similar densities, with moduli of 20.1 MPa, 48.5 MPa, and 109.0 MPa, respectively.

Up to now, only a handful of cross-linked polyamide aerogels, prepared via sol–gel technology, have been studied. Syntheses of PA aerogels generally use low-cost, commercially available materials [20,21], such as p-phenylene diamine (pPDA), m-phenylene diamine (mPDA), terephthaloyl chloride (TPC), or isophthaloyl chloride (IPC), with cross-linkers such as 1,3,5-benzene tricarbonyl trichloride (BTC) [15], or melamine (MA) [22,23]. The resulting PA aerogels had a high density with rigid, stiff backbones. In this paper, a set of 12 flexible polyamide aerogels with a combination of 2,2-dimethylbenzidine (DMBZ) and TPC, cross-linked with tri-isocyanate N3300A, is explored. Desmodur N3300A, a commodity and an inexpensive aliphatic tri-isocyanate, is commercially marketed and used in producing sealants, hardener or two-part adhesive [24,25], polyurethane and polyurethane coatings [26,27], and polyurea aerogels [28,29]. It is also used as a flexible cross-linker to improve silica aerogels’ elasticity and mechanical strength via its urea linkages [30]. N3300A has also been one of the key components in the development of shape memory aerogels [31]. A more flexible monomer DMBZ in contrast with pPDA or mPDA, and the addition of N3300A in place of BTC or MA, was employed in an attempt to lower the rigidity in the polyamide backbone. The experiment was designed with total polymer concentrations in solution varying from 6 to 8 wt.% and with total repeat units, *n*, ranging from 30 to 60 between cross-link sites. The physical, mechanical, and thermal properties, as well as the morphologies of the resulting PA aerogels, will be examined and discussed.

## 2. Results and Discussion

**Figure 1:**
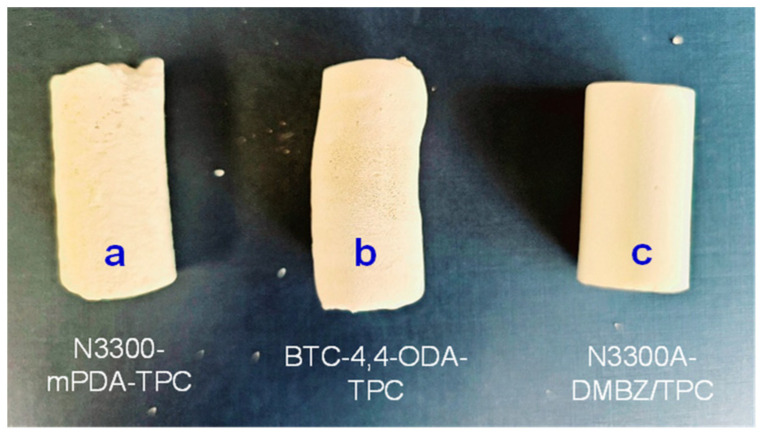
Physical state of cross-linked PA aerogels made with (**a**) mPDA/TPC/N3300A (10 wt.%, *n* = 30), (**b**) ODA/TPC/BTC (10 wt.%, *n* = 30), and (**c**) DMBZ/TPC/N300A (8 wt.%, *n* = 30).

Preliminary research in developing cross-linked polyamide aerogels with formulations consisting of different diamines (i.e., mPDA, pPDA, 4,4-oxydianiline (4,4-ODA), 3,4′-oxydianiline (3,4′-ODA) and DMBZ), diacid chlorides (i.e., IPC and TPC), and cross-linkers (i.e., BTC, TAB, and N3300A) was conducted. Among most products that either precipitated during the reaction of precursors, gelled overnight, which led to highly fragile gels, or never gelled, only a few aerogels were successfully produced and are displayed in Figure 1a–c. These aerogels were composed of mPDA/TPC/N3300A (Figure 1a), 4,4′-ODA/TPC/BTC (Figure 1b), and DMBZ/TPC/N3300A (Figure 1c), with an *n* = 30 at different polymer concentrations (8–10 wt.%). The aerogel shown in Figure 1a (mPDA/TPC/N3300A) was flaky, while the one displayed in Figure 1b (4,4′-ODA/TPC/BTC) was soft and easily disfigured during processing and handling. The aerogel presented in Figure 1c (DMBZ/TPC/N3300A), on the other hand, was smooth and sturdy. Overall, the impact of gelation time, consistency of gel formation, and the physical state of the resulting products indicated DMBZ, TPC, and N3300A as the preferred monomers for this study.

**Scheme 1:**
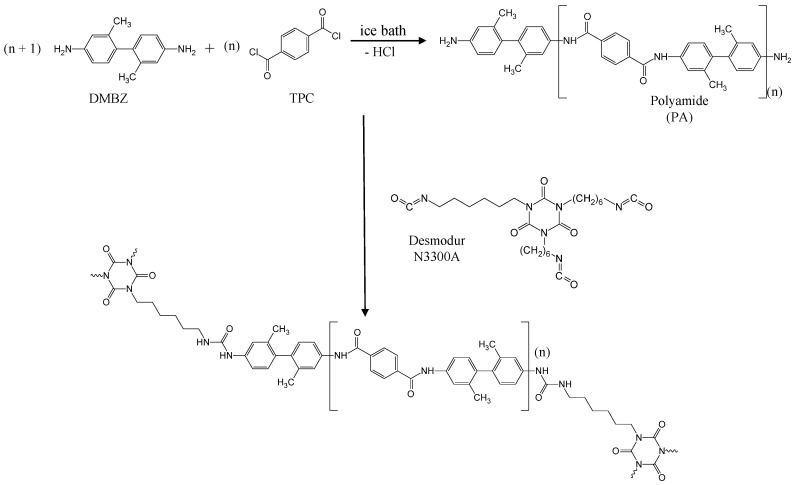
Chemical reaction of a N3300A cross-linked polyamide aerogels.

The chemical reaction of DMBZ and TPC, cross-linked with N3300A, is shown in Scheme 1. Twelve polyamide aerogels were formulated with their corresponding % shrinkage, density, % porosity, BET surface area, Young’s modulus, and final onset decomposition temperatures (T_d_) are listed in Table 1. The cross-linked polyamide was formed with an *n* equivalent of TPC, which was capped with an (*n* + 1) equivalent of DMBZ, followed by a reaction with N3300A.

**Figure 2:**
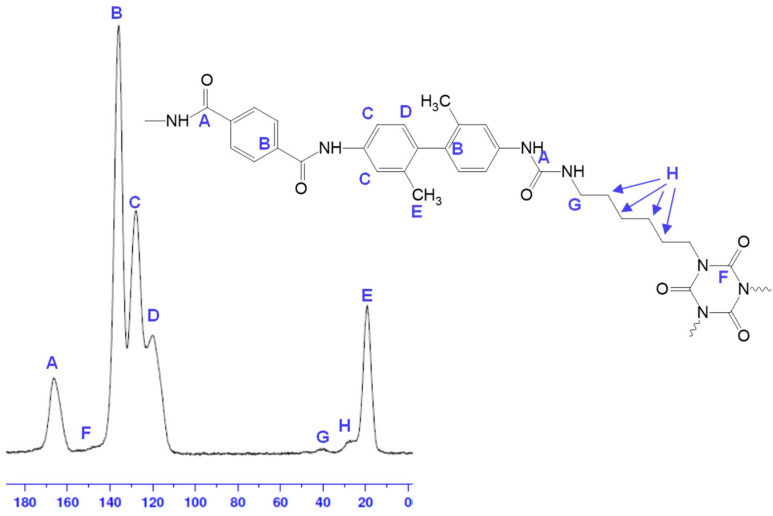
A solid ^13^C NMR of a polyamide aerogel formulated at 6 wt.% total polymer concentration with *n* of 30.

The chemical structure of N3300A cross-linked polyamide aerogels was confirmed using solid ^13^C nuclear magnetic resonance (NMR) spectroscopy. Figure 2 shows the NMR spectrum of an aerogel fabricated at 6 wt.% total polymer and an *n* value of 30 (Run #8). The peak at 168 ppm (A) is assigned to the carbonyls (C=O) from the amide and urea linkages, and the peaks ranging from 120 ppm to 135 ppm are carbons from the aromatic rings (B–D). The peak at 18 ppm is attributed to methyl groups from DMBZ (E) [17,20]. Insignificant signals were detected for N3300A in comparison to the main polyamide chain due to low concentration. Nevertheless, the peak at 148 ppm is assigned to the (C=O) peak from the isocyanurate ring (F) and peaks at 42 ppm and 23 ppm are to the methylene next to the nitrogen (G) and the four carbons in the center (H), respectively, and all are in agreement with other studies conducted by Nguyen et al. [19,30] and Leventis [29].

**Figure 3:**
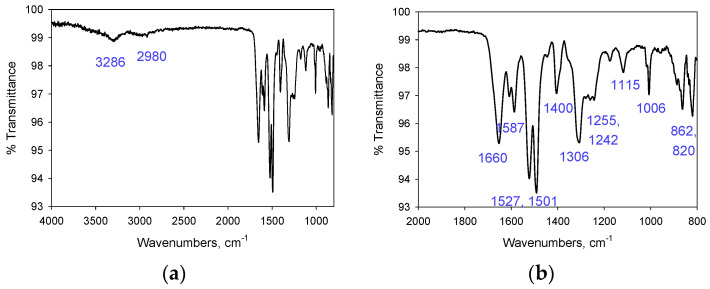
FTIR spectrum of a polyamide aerogel formulated at 6 wt.% total polymer concentration with *n* of 30; (**a**) full scale from 4000 cm^−1^ to 800 cm^−1^ and (**b**) enlarged scale from 2000 cm^−1^ to 800 cm^−1^.

The full-scale FTIR spectrum of a PA aerogel fabricated with *n* of 30 is shown in Figure 3a. Peaks at 3200 cm^−1^ to 3400 cm^−1^ are due to the stretching N-H band from the amide [15] and urea linkages [32], and at 2980 cm^−1^ from -CH_3_ from DMBZ [33] and likely a moiety of the methylene units found in N3300A. Presented in Figure 3b is an enlarged scale from 2000 cm^−1^ to 800 cm^−1^. Peaks observed at 1660 cm^−1^ and 1587 cm^−1^ are carbonyl stretching, and 1527 cm^−1^ and at 1501 cm^−1^ are C=O bending from the secondary amine. The peaks for the isocyanurate ring carbon, typically at about 1690 cm^−1^ [34], were not observed, probably due to the low concentration of N3300A in the formulation. Other peaks generally are from the benzene rings.

**Figure 4:**
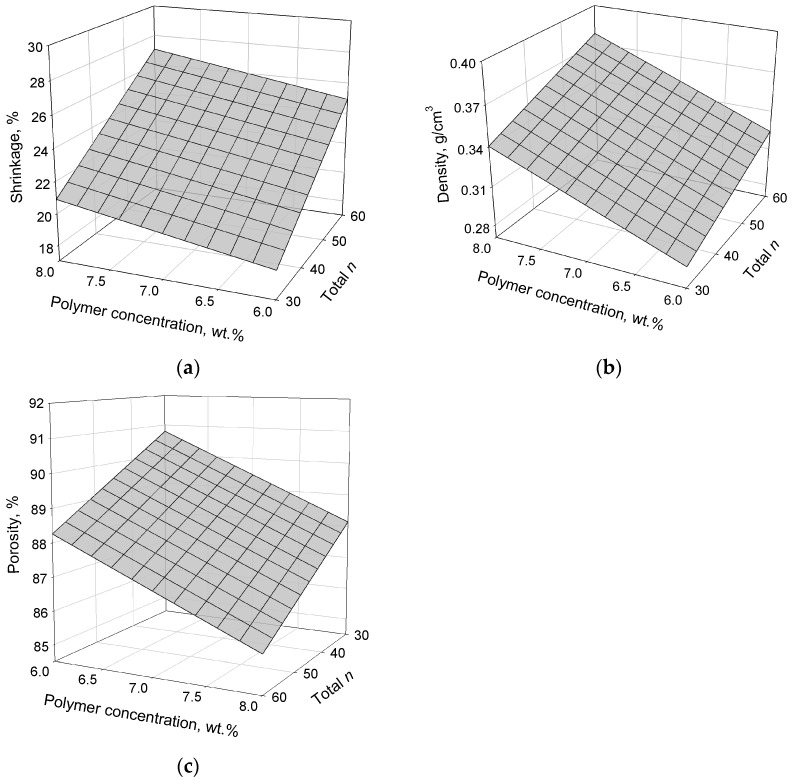
Empirical model for (**a**) shrinkage (%), (**b**) density, and (**c**) porosity (%) vs. polymer concentration and total *n*.

The physical properties of the aerogels are evaluated using empirical models. Graphed in Figure 4a–c is the % shrinkage (R^2^ = 0.83, stdv. = 1.31), density (R^2^ = 0.88, stdv. = 0.01), and % porosity (R^2^ = 0.80, stdv. = 0.77) of the final products versus polymer concentration (wt.%) and repeating units, total *n*, respectively. As observed in Figure 4a, the dimensional change, or % shrinkage, of aerogels was affected by the polymer concentration. The higher polymer concentration may have contributed to the closer packing of oligomer chains, which may have caused stronger intermolecular hydrogen bonding, thus, resulting in higher shrinkage (%) [35]. Density, calculated from the weight of the specimen over its volume, therefore, is a function of shrinkage. Densities were found to increase with increased shrinkage and vice versa (Figure 4b). On the other hand, porosity (%) generally follows the opposite trend seen in density. Presented in Figure 4c is the empirical model of porosity versus polymer concentration and total *n*. It should be pointed out that the porosity plot was graphed with inverse directions of shrinkage and density for a better planar view. Lower porosity was obtained at higher density due to the denser structure of the aerogels.

**Figure 5:**
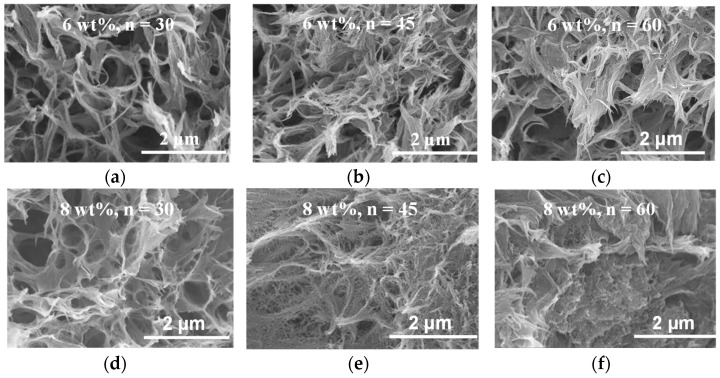
SEM images of the N3300A cross-linked PA aerogels at 6 wt.% with (**a**) *n* = 30 (ρ = 12.1 mg/cm^3^), (**b**) *n* = 45 (ρ = 12.9 mg/cm^3^), (**c**) *n* = 60 (ρ = 13.1 mg/cm^3^), and at 8 wt.% with (**d**) *n* = 30 (ρ = 17.1 mg/cm^3^) (**e**) *n* = 45 (ρ = 16.5 mg/cm^3^) and (**f**) *n* = 60 (ρ = 22.1 mg/cm^3^).

**Figure 6:**
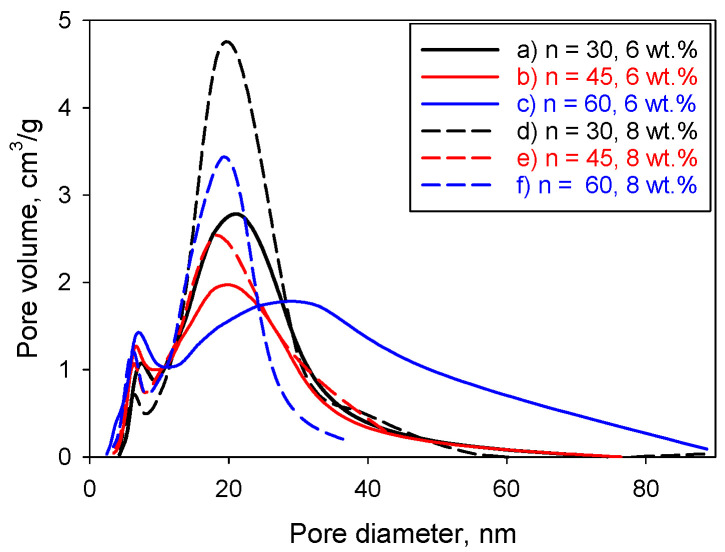
Pore volume vs. pore diameter of the cross-linked PA aerogels at different n values and polymer concentrations.

The morphology of the aerogels was observed via SEM images, displayed in Figure 5a–f. The cross-linked PA aerogels with an *n* of 30 (Figure 5a,d) had a larger pore distribution throughout the specimens, as opposed to those with a longer chain length with a denser structure, as shown in Figure 5b,c,e,f. The difference in pore sizes here is correlated with the porosity (%) as well as shrinkage (%). As found in the physical properties, higher porosity, or lower shrinkage, indicated that polymer strands with shorter chain lengths, or lower *n* between the cross-link sites, had less tendency to collapse, which helped to maintain the pore structure. Their corresponding pore diameter vs. pore volume is plotted in Figure 6a–f for the 6 wt.% and 8 wt.% polymer concentrations with an *n* of 30, 45, and 60. These cross-linked PA aerogels had bi-modal curves with a small pore volume of nanopores, at about 6–7 nm. Except for the aerogel fabricated at 6 wt.% and *n* of 60 (run #4, Table 1), which had a wider pore size distribution over the range from 13 nm to 80 nm (Figure 6c), others exhibited a larger volume of mesopore sizes, from about 18 nm to 40 nm (runs # 1, 6–8, 10) [36]. It was seen that at the higher polymer concentration, 8 wt.%, larger pore volumes and narrower the pore distributions were seen. This outcome may be related to the overall shrinkage of these aerogels. The packing of polymer strands between the cross-linker may also be the cause of the final products having higher volumes with smaller pore sizes compared to their counterparts (6 wt.%).

**Figure 7:**
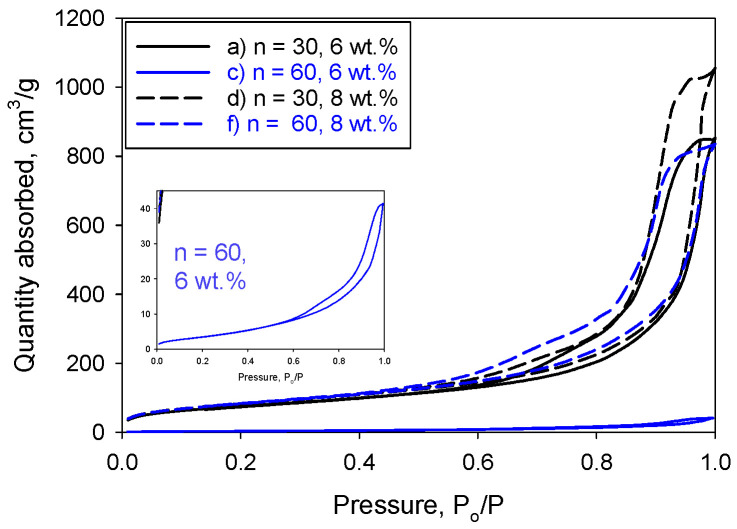
Adsorption and desorption isotherms of the cross-linked PA aerogels at different n values and polymer concentrations.

**Figure 8:**
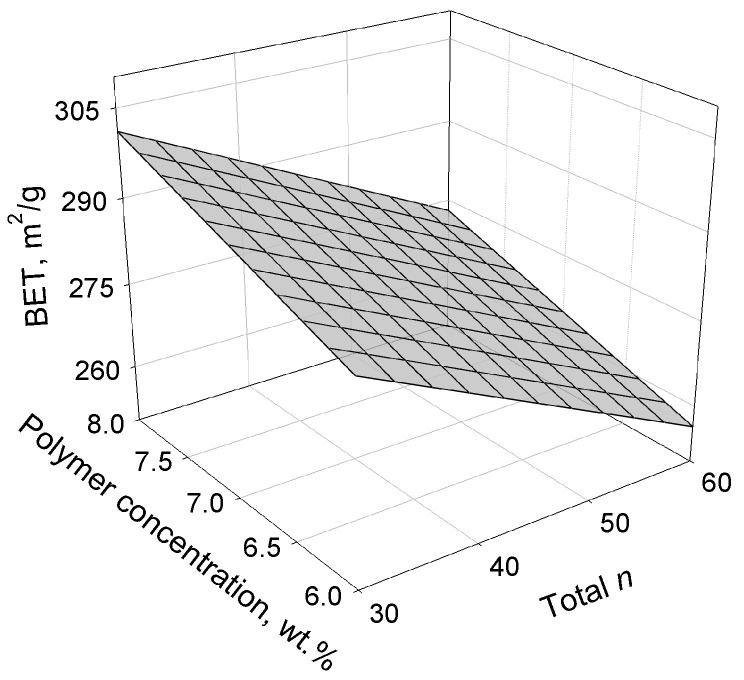
BET surface area of the cross-linked PA aerogels.

Figure 7 shows the nitrogen adsorption and desorption isotherms for selected aerogels, seen in Figure 6. The IUPAC type-IV curves [37] with an H1 hysteresis loop of these aerogels indicated that they were mesoporous, corresponding to the pore size distribution shown in Figure 6. The empirical model of the BET surface area (R^2^ = 0.77, stdv. = 8.57) data in Figure 8 revealed the similar trend seen with density only on the polymer concentration axis, and that a higher polymer content resulted in a higher BET surface area. However, on the repeat unit axis, the higher *n* value led to the lower BET surface area. This outcome was analogous with pore diameters, as represented in Figure 6. The lowest BET surface area was found to be in-line with the aerogel fabricated at the lowest polymer concentration (6 wt.%) and the highest *n* value (60), which had a wide-ranging pore size and the lowest pore volume (Figure 6c). The highest BET surface area, modeled from the pool of data, was achieved for aerogels made with 8 wt.% polymer concentrations and an *n* of 30 (Figure 6d), and shows the highest volume of mesopores among all of the samples.

**Figure 9:**
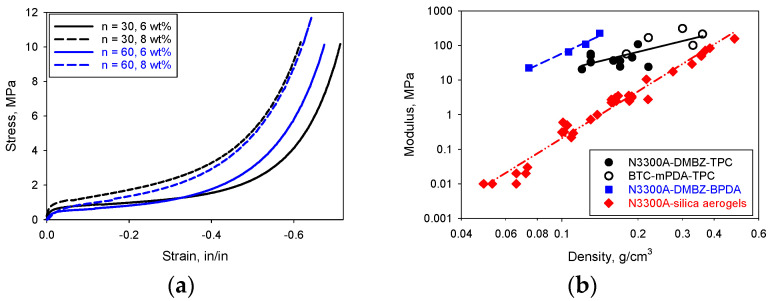
(**a**) Typical stress–strain curves from compression test of N3300A-cross-linked PA aerogel; (**b**) log–log plot of density vs. Young’s modulus of N3300A-DMBZ-TPC (closed symbol), and BTC-mPDA-TPC (open symbol) [15] polyamide aerogels, N3300A-DMBZ-BPDA polyimide aerogels [19], and N3300A-reinforced silica aerogels [30].

The mechanical properties of the N3300A-cross-linked PA aerogels were performed on cylindrical specimens under compression. Polyamide aerogel monoliths were prepared according to the standard test method ASTM D695-10 [38]. Typical stress–strain curves are plotted in Figure 9a for the aerogels with an *n* of 30, 6 wt.% (run #8), *n* of 30, 8 wt.% (run #9), *n* of 60, 6 wt.% (run #4), and *n* of 60, 8 wt.% (run #1). Young’s modulus is measured at the initial slope of their elastic region and is a function of density. Illustrated in Figure 9b is a graph of power law dependencies between density and the modulus of polyamide (PA), N3300A-polyimide (PI), and N3300A-reinforced silica aerogels, with the exponent b[1] of 1.78, 2.34, and 4.51, respectively. For PA aerogels, analysis was carried out on the pooled data collected from the N3300A-DMBZ-TPC (*n* of 30–60) set in combination with those from the previously reported BTC-mPDA-BTC (*n* of 20–40) set [15]. The N3300A-PA aerogels exhibited lower moduli (b[1] = 1.78) compared to the N3300A-PI aerogels (b[1] = 2.34). The difference in modulus between the N3300A cross-linked PA and the N3300A cross-linked PI was perhaps due to the more flexible DMBZ-TPC bonds than the DMBZ-BPDA bonds. As also found, replacing mPDA with a less rigid aromatic diamine DMBZ, and the cross-linker BTC with an aliphatic N3300A, resulted in lower density and lower moduli, thus reflecting a more flexible structure overall. Silica aerogels, having a pearl-necklace feature, are known to be fragile [39,40] compared to most polymer aerogels. Hence, it is understandable that the analogous mechanical strength of silica aerogel in general is expected to be lower at similar densities. However, this weakness at the neck region could be enhanced by conformally coating their skeletal framework with polymers [39,40]. For example, the compression modulus of a polymer-silica hybrid aerogel made of methyltrimethoxysilane (MTMS) and bis(trimethoxysilylpropyl)amine (BTMSA), reinforced with N3300A, greatly increased with increasing density [30]. With the highest b[1] of 4.51 among all three types of aerogels, the N3300A-silica aerogel became stronger with increasing density, due to the densification of the silica backbones. Although N3300A was found to have a slight effect that governed the moduli, in this case, the reinforced silica aerogels with N3300A as a conformal coating were found to yield higher elasticity with lower unrecovered strain (%) compared to their native counterparts [30]. It is interesting that the choice of N3300A cross-linker could be used to improve the flexibility of aerogels if needed, especially at a fraction of the cost of BTC and TAB. The increase in modulus as density increased for all three materials was possibly influenced by the higher shrinkage (%) and by the fact that all these aerogels had a higher density.

**Figure 10:**
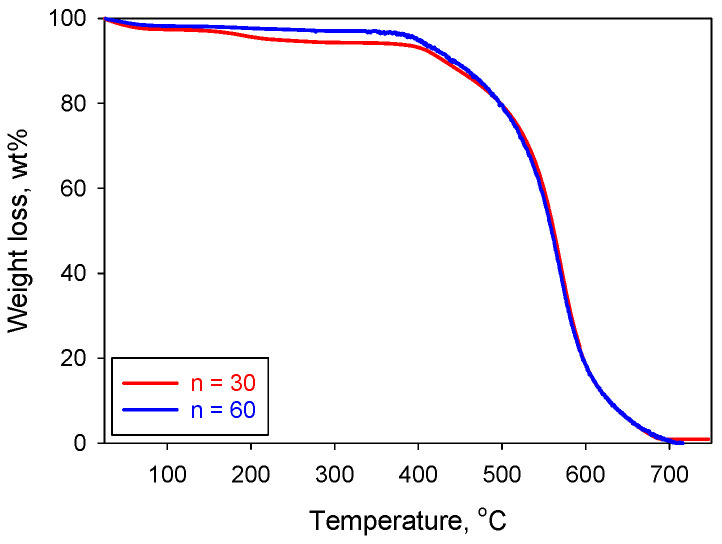
TGA curves of N3300A cross-linked PA aerogels at *n* = 30 and *n* = 60.

**Figure 11:**
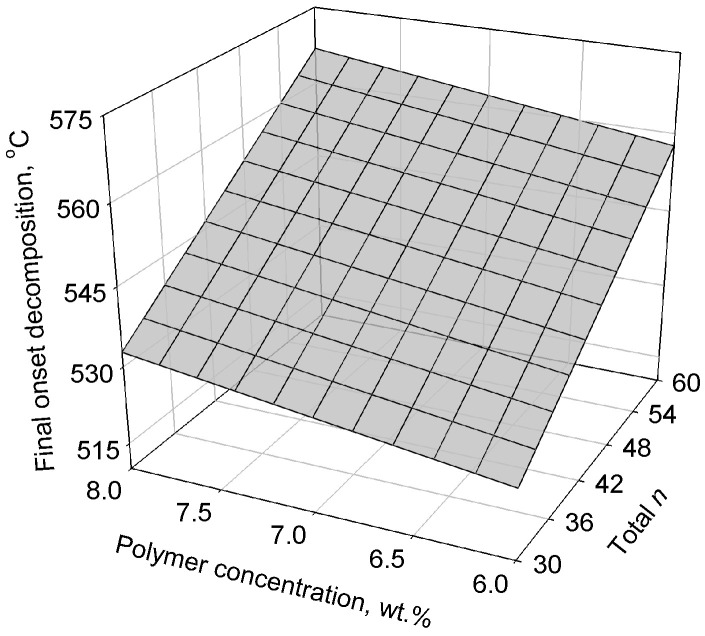
Empirical model of final onset decomposition temperature of N-3300A cross-linked polyamide aerogel.

The thermal stability of the cross-linked PA aerogels from this study was determined using TGA and measured as the onset of decomposition temperature, T_d_, in air. The TGA curves of the polymer over a range of temperatures up to 800 °C, with *n* of 30 and *n* of 60, are shown in Figure 10. Polyamides are hygroscopic; therefore, the initial weight loss event occurring before 195 °C may have been due to the loss of water and was not quantified. The weight loss seen from about 195 °C to 400 °C was likely due to the degradation of the aliphatic chains from the cross-linker N3300A [41]. A higher % weight loss was seen with increasing amounts of the cross-linker. Weight loss from N3300A, However, was not apparent, likely due to its relatively low concentration in the formulations. The complete degradation was mostly due to the breaking of amide bonds, in addition to the urea linkages at the cross-link sites and began around 522 °C (Table 1). The empirical model of the final onset of decomposition temperature, T_d_, vs. polymer concentration and total *n*, is graphed in Figure 11. Following a similar trend observed in density, PA aerogels exhibited a lower T_d_ at lower polymer concentrations wt.%) and lower *n*, an indication of having a lower content of amide bonds. In contrast, a higher T_d_ was obtained when PA aerogels were fabricated at higher wt.% and higher *n*, which consisted of a higher number of amide bonds. As discussed previously, the alignment of the oligomer chain length between N3300A, with a higher tendency of intermolecular hydrogen bonding, may have contributed to the thermal stability of the aerogels.

## 3. Conclusions

A series of 12 flexible polyamide aerogels with a backbone composed of TPC and DMBZ, cross-linked with an inexpensive, commercially available tri-isocyanate N3300A, was fabricated. The synthetic route was straight forward, and their properties were tunable by modifying the 3-D structure with polymer concentrations ranging from 6 to 8 wt.% and repeating units, *n*, from 30 to 60. Unlike the flaky or disformed PA aerogels made with mPDDA or ODA (Figure 1a or Figure 1b), respectively, the DMBZ-containing polyamide aerogels maintained their uniform, good shape after fabrication (Figure 1c), even at a higher shrinkage of up to 27%. They were non-fragile, sturdy, and easy to handle. Compared to the polyamide aerogels constructed from rigid monomers like mPDA and BTC, substituting these with DMBZ and N3300A resulted in lower densities and lower moduli than other materials cross-linked or reinforced by N3300A, thereby reflecting higher flexibility. It was also found that the thermal stability of the cross-linked polyamide aerogels was greater at higher polymer concentration and higher *n*, due to the higher content of amide bonds between cross-link sites and vice versa. This phenomenon was likely the result from the polymer chain alignment between N3300A with a high tendency to form intermolecular hydrogen bonds.

## 4. Materials and Methods

### 4.1. Materials

2,2′-Dimethylbenzidine (DMBZ) was purchased from Wakayama Seika Kogya Com., Ltd., Wakayama, Japan, and tetraphthaloyl chloride (TPC), N-methyl pyrrolidinone (NMP), and acetone were obtained from Sigma Aldrich. Desmodur N3300A was purchased from Bayer Material Science and was used as received.

### 4.2. Synthesis of N3300A Cross-Linked Polyamide Aerogels

**Scheme 2:**
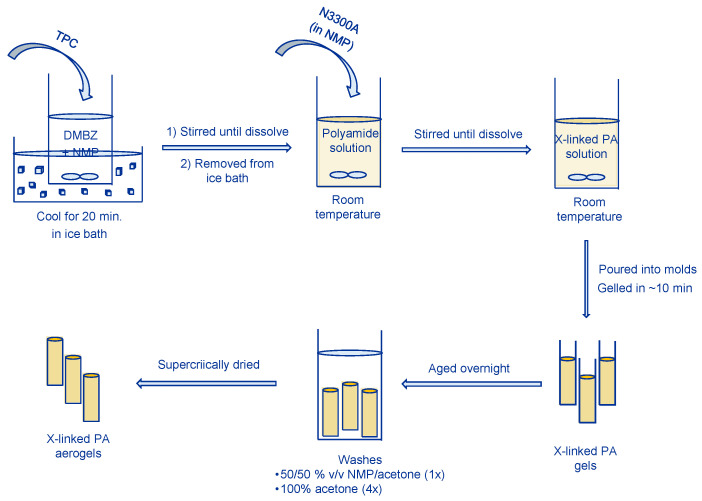
Schematically synthetic steps in fabricating N3300A cross-linked polyamide (or x-linked PA) aerogels.

The fabrication of the N3300A cross-linked polyamide aerogels were prepared as demonstrated in Scheme 2. Following is an example of preparing a cross-linked PA aerogel, formulated at 8 wt.% of total polymers with an *n* value of 60 (run # 1). A solution of 2.4577 g (11.5 mmol) of DMBZ in 40 mL of NMP was chilled in an ice bath for 20 min, then 2.2934 g (11.3 mmol) of TPC was added. Once dissolved, the mixture was stirred for another 5 min before being removed from the ice bath and was brought up to room temperature. An amount of 0.0727 g (0.126 mmol) N3300A, dissolved in 4.75 mL NMP, was added to the polyamide solution, and the mixture was stirred thoroughly for another 5 min. The N3300A cross-linked polyamide solution was poured into cylindrical molds. Gelation occurred within 10 min. The wet gels were aged at room temperature overnight. The hydrochloric acid (HCl), generated from the reaction of DMBZ and TPC, and NMP in the gels were removed by solvent exchanging with one 50:50 v/v% ratio of NMP/acetone solution, followed by four rinses with neat acetone, twice daily. All cross-linked polyamide (PA) gels were supercritically dried using CO_2_ extraction in an Accudyne multivessel automated system. The resulting aerogels were further dried in an oven at 80 °C under full vacuum for 12 h to remove any moiety of water. It should be noted that gelation time of gels depended on the formulations designed. Solution with higher polymer concentrations (wt/wt%) and/or shorter chain lengths (lower *n* values) gelled faster, within 10 min, than those having lower concentrations and/or longer *n*, which could be up to 20 min.

### 4.3. Instrumentation

The cross-linked polyamide aerogels were dried via liquid CO_2_ supercritical extraction using an Accudyne multi-vessel automatic system. The products were further outgassed at 80 °C under full vacuum overnight. NMR spectra were obtained using a Bruker Avance 300 spectrometer. The skeletal densities (ρ_s_) of the aerogels were determined using a Micromeritics Accupyc 1340 helium pycnometer. The Burnauer–Emmett–Teller (BET) surface areas and pore distribution were measured using a Micromeritics ASAP 2020 chemisorption instrument with sample weights in the range of 0.010 mg to 0.15 mg, which were further degassed at 85 °C for another 12 h under full vacuum. Thermal gravimetric analysis (TGA) was performed on a TA model Q500 from room temperature to 750 °C at a temperature ramp rate of 10 °C/min under air. Morphology structure images were taken using a Hitachi S-4700-11 field emission scanning electron microscope (SEM).

### 4.4. Mechanical Characterization

The compression test was carried out on cylindrical monoliths according to ASTM D695-10. The monoliths, with both ends sanded to be parallel, were prepared with a ratio from 1.25 to 1.50 of length to diameter.

### 4.5. Physical Characterizations

The bulk density (ρ_b_) of the cylindrical monoliths was calculated from the weight divided by the volume (calculated from manually measuring the cylinder dimensions). The shrinkage (%) of a monolith was measured by the difference between the diameter of the mold (normally 20 cm) and the final diameter of the final product. Measurements of density and shrinkage were averaged from data collected from 3 aerogel cylinders. The porosity (%) of an aerogel was determined from the bulk and skeletal densities using Equation (1)

Porosity % = (1 − ρ_b_/ρ_s_) × 100 (1)

### 4.6. Analyses

Empirical models of density, shrinkage, porosity, BET, and final onset decomposition temperature vs. polymer wt.%, and the total *n* of the N3300 cross-linked DMBZ/TPC aerogels (Table 1) were analyzed using the Design-Expert 13 software from Stat-Ease.

## Figures and Tables

**Table 1:** Formulation and properties of N3300A cross-linked polyamide aerogels.

Run #	Polymer Conc. (wt.%)	Repeat Unit (*n*)	Density (mg/cm^3^)	Shrinkage (%)	Porosity (%)	BET Surface Area (m^2^/g)	Young’s Modulus (M(Pa)	Decomposition Temperature of Polyamide (°C)
1	8	60	22.1	26.46	82	308	24.18	570
2	6	40	13.3	21.47	90	284	50.89	540
3	8	40	19.8	24.48	84	295	109.02	554
4	6	60	13.1	20.79	89	300	32.89	522
5	7	50	16.2	22.63	87	272	36.39	531
6	6	45	12.9	21.16	90	265	27.39	537
7	8	45	16.5	19.14	88	269	36.05	538
8	6	30	12.1	19.47	91	282	20.6	522
9	8	30	16.4	20.03	88	309	37.29	541
10	8	30	17.1	19.85	88	291	(-)	524
11	8	50	18.9	26.63	86	312	45.39	555
12	6	50	16.5	26.47	88	297	24.71	540

(-) not measured.

## Data Availability

The original contributions presented in the study are included in the article. Further inquiries can be directed to the corresponding author.
